# Chronic stable recording of physiologically evoked neural activity in a mixed autonomic nerve in awake, freely moving normal and experimental cystitis rats

**DOI:** 10.1063/5.0336334

**Published:** 2026-08-14

**Authors:** Sophie C. Payne, Christopher Bowman, Peregrine B. Osborne, Jerico Matarazzo, Janet R. Keast, James B. Fallon

**Affiliations:** 1 Bionics Institute, Melbourne, Victoria 3002, Australia; 2 Medical Bionics Department, University of Melbourne, Melbourne, Victoria 3010, Australia; 3 Swinburne University of Technology, Melbourne, Victoria, Australia; 4 Department of Anatomy and Physiology, University of Melbourne, Melbourne, Victoria 3010, Australia

## Abstract

Closed-loop control over bladder function, for conditions such as neurogenic bladder dysfunction, requires real-time monitoring of bladder state. Here, we assessed the ability of a novel recording and analysis technique to extract neural activity from awake rats as a quasi-real-time feedback biomarker of bladder fullness in normal and chronic cystitis models. Adult male rats (*n* = 12) were implanted with a four-electrode planar array and the bladder instrumented for continuous-flow cystometry over 2 weeks. In three rats, bladder inflammation was induced using cyclophosphamide (CYP, 75–100 mg/kg, i.p.). Neural recordings were made during awake cystometry, and extracted Aδ afferent activity was correlated with bladder pressure. In control rats (*n* = 9), there was a strong, stable correlation of integrated Aδ activity with bladder pressure during filling at 1 week (*R*^2^: 0.55 ± 0.04) and 2 weeks (*R*^2^: 0.57 ± 0.04; *p* > 0.05). In CYP-cystitis rats (days 9, 11, and 14), there was no difference in this correlation (week 2: 0.54 ± 0.031; *p* > 0.05). Taken together, data suggest the recording and analysis technology remained stable in awake, freely moving normal rats and during 2 weeks of chronic implantation. Furthermore, data show potential that abnormal activation of nociceptive afferents in cystitis rats, which causes an increase in baseline activity, does not weaken correlation of Aδ activity with bladder pressure. In conclusion, this feasibility study is a promising first study step for the translation of closed-loop technology for bladder control for neurogenic bladder dysfunction.

## INTRODUCTION

Neurogenic bladder dysfunction commonly occurs during spinal cord injury, multiple sclerosis, and diabetes, where visceral nerves controlling the bladder are impaired, causing issues with bladder emptying or urine storage (Phé *et al.*, 2016; Wyndaele, 2016; Ginsberg *et al.*, 2021; and Song *et al.*, 2022). Standard treatments, such as intermittent self-catheterization are invasive and socially unpleasant, while pharmacological medications (i.e., anticholinergic blockers) are often associated with unpleasant cognitive side effects and hypotension (Serlin *et al.*, 2018; Verpoorten and Buyse, 2008; and Phé *et al.*, 2016). As such, in recent years the use of electrical implantable neuromodulation devices to control bladder function has gained interest as an alternative treatment strategy for neurogenic bladder dysfunction (Bartley *et al.*, 2013; Oh *et al.*, 2024; Krhut *et al.*, 2025; and Cracchiolo *et al.*, 2021). Sacral nerve stimulation (SNS), which targets visceral afferent spinal roots, is FDA-approved for urinary urge incontinence (Brazzelli *et al.*, 2006; Krhut *et al.*, 2025). However, many users report device-related perineal pain during SNS (Everaert *et al.*, 2000). Neuromodulation of the pelvic splanchnic nerves in humans could have applications for neurogenic bladder dysfunction. This is supported by preclinical evidence from studies of the homologous pelvic nerve in rodent models, which have established that open- and closed-loop neuromodulation devices can be used to control and externally monitor activity of the bladder (Keast, 2006; Crook and Lovick, 2017; Langdale *et al.*, 2017; 2020; Brouillard *et al.*, 2018; and Payne *et al.*, 2020; 2023). For example, we and others have reported that pelvic nerve neuromodulation with different electrical stimulation parameters can either initiate an involuntary voiding reflex or arrest/delay a voiding reflex during saline cystometry in rats or cats (Crook and Lovick, 2017; Langdale *et al.*, 2017; 2020; Brouillard *et al.*, 2018; and Payne *et al.*, 2020). Current neuromodulation systems for bladder control are “open-loop” and provide fixed levels of stimulation that are not specific to the physiological state of the bladder (Park *et al.*, 2018; Oh *et al.*, 2024). The next generation of neuromodulation devices for bladder control aims to provide “closed-loop” stimulation, in which electrical stimulation is applied to either initiate or arrest a void, depending on the physiological state of the bladder (Park *et al.*, 2018; Ouyang *et al.*, 2022; and Oh *et al.*, 2024). However, a critical requirement of a device to control bladder function is reliable real-time feedback on the physiological state of the bladder in order for appropriate, timely stimulation to be delivered to either initiate or arrest a void (Oh *et al.*, 2024).

Our laboratory has previously demonstrated a novel recording and analysis technology for the monitoring of pelvic nerve Aδ afferent fiber activity, which are fast-conducting, thinly myelinated sensory fibers that detect mechanical stretch from bladder filling (de Groat and Yoshimura, 2009) during saline cystometry in urethane-anesthetized rats (Payne *et al.*, 2023). In this study, a two-paired longitudinal interface nerve electrode (LINE) array was placed perpendicular over the pelvic nerve to allow recording of physiologically evoked neural activity recorded from the mixed autonomic (pelvic) nerve. Neural activity recorded from the LINE array are coupled with a novel signal processing method (Fallon, 2019) that runs sliding cross correlation analyses between two pairs of recordings (Payne *et al.*, 2023). By comparing the timing and direction of pelvic neural activity between two pairs of recording electrodes, the type (i.e., afferent vs efferent) of neural activity is reflected by the polarity, and the class (i.e., Aδ or C-fiber) of neural activity is indicated by the timing. Using this novel recording and analysis technique, we have shown in an anesthetized preparation that integrated Aδ afferent activity extracted from the pelvic nerve correlated with bladder pressure during cystometry-induced voiding (Payne *et al.*, 2023). Therefore, a next step to translating the technology into the clinic for closed-loop bladder control is to conduct a feasibility study that assesses how stable the extraction of Aδ afferent activity is to factors (e.g., fibrotic changes in neural interfaces) arising in awake, freely moving chronically implanted animals. As a second aim, we assessed the stability of integrated Aδ afferent activity correlation with bladder pressure in animals challenged with cyclophosphamide (CYP) to induce cystitis and an increase in Aδ afferent and C-fiber nociceptor activity. CYP is a well-established bladder inflammatory agent that is metabolized into acrolein, a toxic compound that damages the bladder's protective lining, to cause rapid voiding (Arms and Vizzard, 2011).

**FIG. 1.**
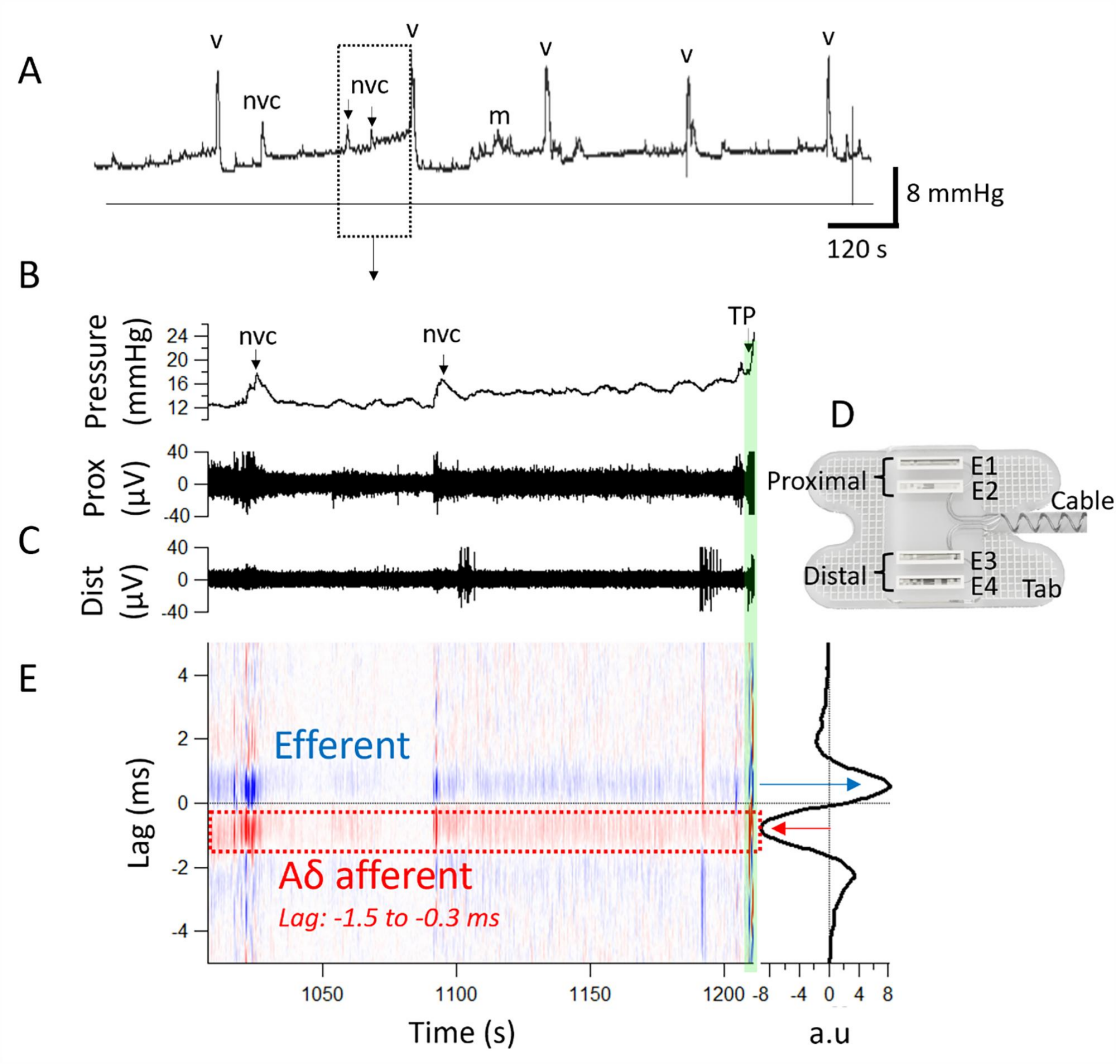
Representative example of cystometry and cross correlation analysis of physiologically evoked pelvic nerve activity in an awake rat at 2 weeks following implantation surgery. (a) Saline-induced cystometrogram shows voiding (v), non-voiding contractions (nvc), and movement artifact (m). (b) A representative waveform of a pre-void filling phase shows non-voiding contractions (nvc) and threshold pressure (TP, indicated by green shading). (c) Differential recordings were taken from proximal electrodes (proxi, E1–E2) and distal electrodes (dist, E3–E4). (d) Schematic drawing of the electrode array implanted perpendicular onto the pelvic nerve. The distance between bipolar proximal (E1–E2) and distal (E3–E4) pairs was 3.25 mm, and the subcutaneous return electrode (not shown) was located on the cable. (e) The output of the cross correlation between recordings (prox, dist) is a heatmap, with darker colors representing more neural activity. Blue represents negative correlations, and red represents positive correlations. The time lag of the peak correlation indicates type of neural activity (fast vs slow), while the sign of the lag (positive or negative) indicates class (sensory vs motor). The accepted lag of Aδ afferents was −1.5 to −0.3 ms (Sengupta and Gebhart, 1994b). Here, we show extraction of a negative lag of −0.5 ms that equates to 6.5 m/s, indicative of fast Aδ afferent activity, and a positive lag indicative of preganglionic efferent activity.

Recording Aδ afferent activity associated with “threshold pressure” (TP), the level of intravesical bladder pressure reached that triggers the contraction of the detrusor muscle, is potentially particularly informative for closed-loop systems (Park *et al.*, 2018; Oh *et al.*, 2024). When threshold pressure is reached, the stretch of bladder wall tissue has reached a point where myelinated, tension-detecting Aδ afferent activation overrides the bladder's inhibitory storage mechanisms to initiate voiding (de Groat and Yoshimura, 2009). As such, appropriate neuromodulation intervention applied at this moment could either arrest an unwanted void for incontinence or initiate a voiding cycle for urinary retention. Therefore, the central hypothesis of this study is that Aδ afferent activity during passive filling and at threshold pressure will correlate with bladder pressure. To test this hypothesis, this study used awake adult male rats implanted with a minimally invasive LINE array overlying the pelvic nerve and a bladder catheter used to measure bladder pressure during continuous infusion of saline [Figs. 1(a) and 1(b)] (Payne *et al.*, 2020). We correlated the extracted Aδ afferent activity [lag: −0.3 to −1.5 ms; conduction velocity: 2.2 to 10.8 m/s; Fig. 1(c)] with bladder pressure around threshold pressure over a 2-week chronic implantation period in normal and CYP-challenged rats.

## RESULTS

### Stability of integrated neural activity correlation with bladder pressure in control rats

#### Urodynamic parameters of control, chronically implanted and catheterized rats

A key objective of this study was to develop a real-time neural-based biomarker that accurately informs upon bladder pressure. Therefore, it was critical to confirm that the chronically implanted array and catheter did not affect the urodynamic cycle (Fig. 1). Stability of urodynamic parameters over Time (1 vs 2 weeks) in control rats (*n* = 9) was assessed using a parametric paired Student t-test (two-tailed) (Table I). Data show there were no changes in intervoid interval (IVI) [Fig. 1(a)] between weeks 1 and 2 (t_(8)_ = 1.8; *p* = 0.116; Table I). Similarly, there were no changes over time in TP (t_(8)_ = 1.7; *p* = 0.122), opening pressure (OP) (t_(8)_ = 0.90; *p* = 0.393), and closing pressure (CP) (t_(8)_ = 0.22; *p* = 0.83; Table I).

**TABLE I.** Urodynamic parameters during continuous saline cystometry in implanted control or CYP-challenged rats. Urodynamic data for control rats show mean ± SEM and relative change in pressure from baseline pressure. Differences between 1 and 2 weeks were assessed using a parametric Student t-test. Data for CYP rats are reported as median and interquartile range (IQR), and differences between 1 week (pre-CYP) and 2 weeks (post CYP) assessed using a non-parametric Mann–Whitney U test. Significant differences (indicated by “*” bold/underlined text) between weeks 1 and 2 with control and CYP-challenged rat urodynamic parameters were indicated as “*” and significance was accepted as *p* ≤ 0.05.

Urodynamic parameters (units)	Week 1	Week 2
	Control rats (mean ± SEM)	
Subjects	9	9
No. voids	94	92
Intervoid interval (min)	9.6 ± 0.6	11.9 ± 1.5
Relative threshold pressure (mm Hg)	7.2 ± 1.6	5.2 ± 0.9
Relative opening pressure (mm Hg)	19.5 ± 2.3	18.4 ± 2.0
Relative closing pressure (mm Hg)	29.3 ± 4.6	30.0 ± 6.0
	CYP-challenged rats (median, IQR)	
Subjects	3	3
No. voids	21	36
Intervoid interval (min)	11 (8.4–11.6)	**4.5 (2.5–4.9)** *
Relative threshold pressure (mm Hg)	3.9 (3.7–4.6)	**2.0 (1.3–3.5)** *
Relative opening pressure (mm Hg)	14.2 (13.4–18.6)	12.5 (11.1–16.2)
Relative closing pressure (mm Hg)	15.8 (7.7–26.7)	23.6 (10.1–37.2)

#### Correlation of integrated neural activity with bladder pressure

**FIG. 2.**
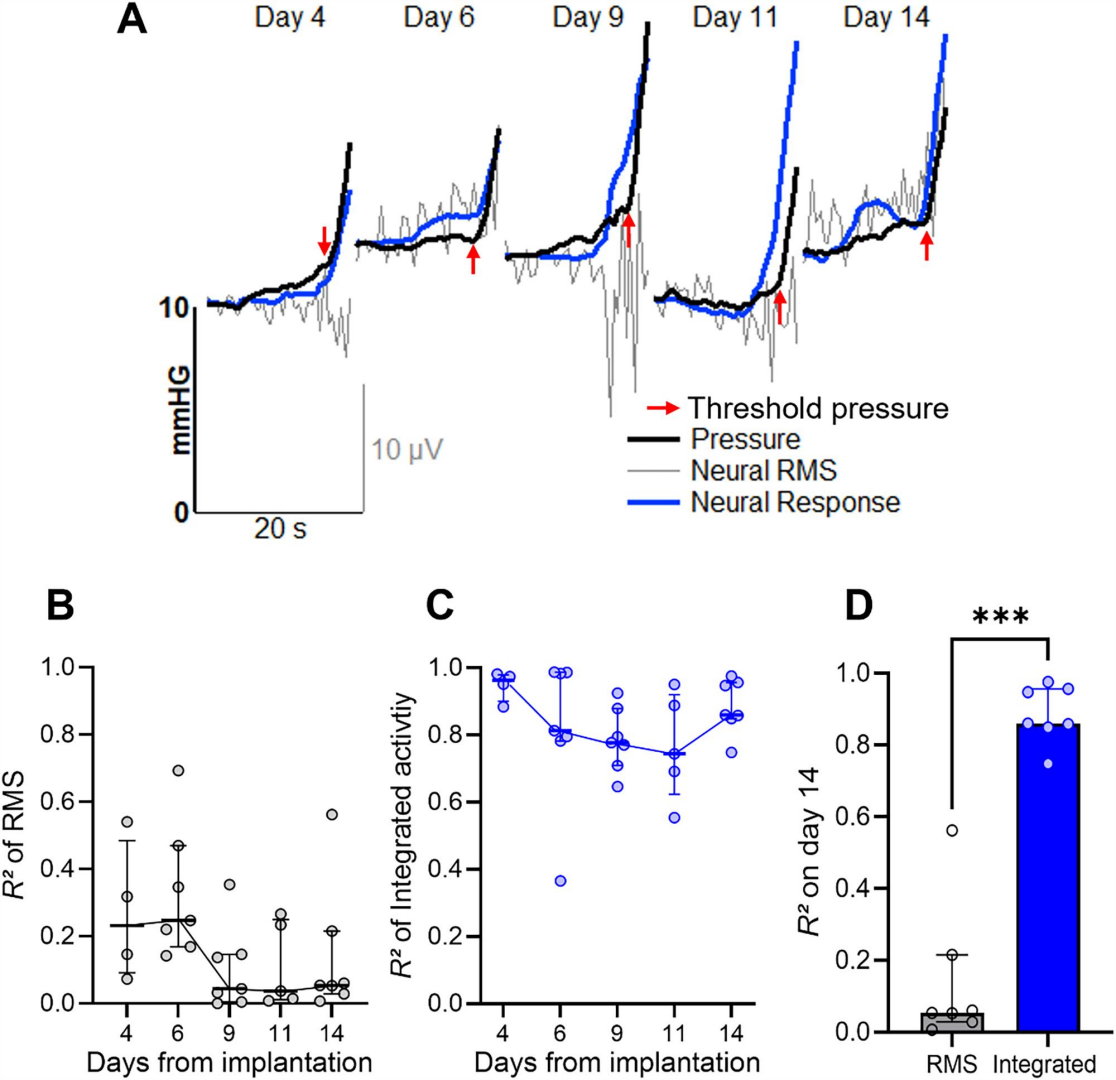
Pressure and neural activity during 2 weeks of saline-induced voiding in a control catheterized rat implanted with a pelvic nerve electrode array. (a) Representative pressure (black trace), RMS neural activity (gray trace), and integrated Aδ activity (−1.5 to −0.3 ms, blue trace) were averaged from 4 to 7 saline-induced voids and overlaid on days 4, 6, 9, 11, and 14 following implantation surgery. The threshold pressure (TP) is indicated in each example by a red arrow. (b) *R*^2^ of neural RMS and pressure remained unchanged (H_(4)_ = 9.03; *p* = 0.060; *n* = 30 voids from *n* = 1 control rat) over implantation time. (c) *R*^2^ of integrated Aδ activity and pressure remained unchanged (H_(4)_ = 7.75; *p* = 0.101; *n* = 30 voids from *n* = 1 control rat) over implantation time. (d) On day 14 following implantation, *R*^2^ of integrated Aδ activity was significantly higher (*p* = 0.016) than *R*^2^ of RMS. Data in (b)–(d) show individual data from a void, median ± interquartile range of *R*^2^ from 4 to 7 consecutive voids (*n* = 30 voids total) of one individual control implanted rat. A Kruskal–Wallis (b) and (c) or Wilcoxon (d) test was performed, and significance was accepted as *p* ≤ 0.05.

A critical urodynamic parameter for neuromodulation devices for bladder control is threshold pressure (TP), which is the moment the detrusor muscle first contracts to initiate a voiding sequence (Fraser *et al.*, 2020). The period prior to TP represents when neural activity is most informative about bladder state and the potential moment when neuromodulation therapies could intervene (Park *et al.*, 2018). As such, analysis of bladder pressure and neural activity was limited to stages of filling prior to and at TP [Fig. 2(a), indicated by red arrows]. The average of 4–7 saline-induced voids was used to represent bladder pressure [Fig. 2(a), black traces], sliding root mean square (RMS) [Fig. 2(a), gray traces], and integration of the extracted afferent activity [Fig. 2(a), blue traces] from a representative rat measured on days 4, 6, 9, 11, and 14 following implantation surgery are shown in Fig. 2(a).

As *R*^2^ data are typically not normally distributed, a non-parametric Kruskal–Wallis was performed to assess changes in *R*^2^ of RMS or integrated extracted Aδ activity during chronic implantation in *n* = 1 control implanted rat. Analysis showed there were no significant changes over time for the *R*^2^ of RMS [H_(4)_ = 9.03; *p* = 0.060; *n* = 30 voids; Fig. 2(b)] or the *R*^2^ of the integrated extracted Aδ activity [H_(4)_ = 7.75; *p* = 0.101; Fig. 2(c)]. Differences between the two analysis methods of *R*^2^ on day 14 following chronic implantation were assessed using a non-parametric paired Wilcoxon signed-rank test and showed the *R*^2^ of integrated activity was significantly higher (median: 0.86, *n* = 7 pairs; W = 28, *p* = 0.016) than the *R*^2^ of RMS [median: 0.054; Fig. 2(c)].

### Stability of integrated neural activity correlation with bladder pressure in rats following CYP injection

#### Urodynamic changes following CYP injection

**FIG. 3.**
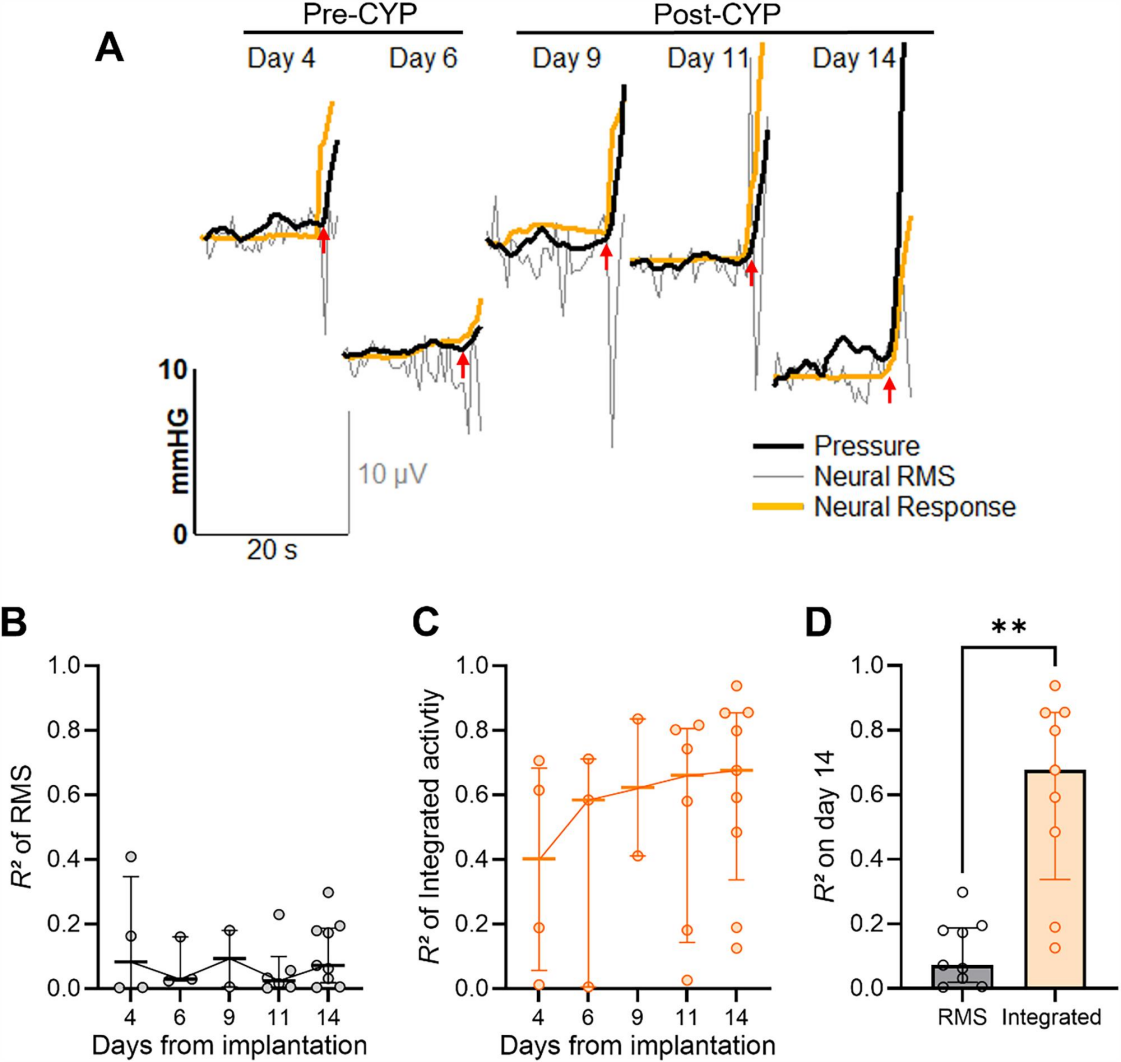
Pressure and neural activity during 2 weeks of saline-induced voiding in a rat challenged with CYP. (a) Representative example, pressure (black trace), RMS activity (gray traces), and integrated Aδ activity (−0.3 to 1.5 ms, orange trace) were averaged from 2 to 9 saline-induced voids. Pre-CYP or baseline voiding occurred on days 4 and 6, while CYP affected voids occurred on days 9, 11, and 14 following implantation surgery. Threshold pressure (TP) is indicated by a red arrow in each example. (b) *R*^2^ of neural RMS and pressure remained unchanged (H = 1.30; *p* = 0.862; *n* = 24 voids from *n* = 1 CYP-challenged rat) over implantation time. (c) *R*^2^ of integrated Aδ activity and pressure remained unchanged (H = 2.68; *p* = 0.613; *n* = 24 voids from *n* = 1 CYP-challenged rat) over implantation time. (d) On day 14 following implantation, *R*^2^ of integrated Aδ activity was significantly higher (*p* = 0.004) than RMS of *R*^2^. Data in (b)–(d) show individual data from a void, median ± interquartile range of *R*^2^ from consecutive voids of one individual CYP-challenged rat. A Kruskal–Wallis (b) and (c) or Wilcoxon (d) test was performed and significance accepted as *p* ≤ 0.05.

Recording Aδ afferent activity associated with threshold pressure (TP) was assessed in rats challenged with CYP, an established model of inflammatory cystitis that results in an increase in Aδ afferent and C-fiber nociceptor activity (Fowler, 2002; Arms and Vizzard, 2011; and Kuga *et al.*, 2016; 2017) (Fig. 3).

In a subset of rats, baseline cystometry recordings were taken during week 1, an injection of CYP was administered, and changes in urodynamic parameters were measured in week 2. As such, a Mann–Whitney U test was conducted to determine if there were differences in urodynamic parameters between weeks 1 and 2 in CYP-challenged rats (*n* = 3; Table I). Data show IVI in week 2 (*p* = 0.05) significantly decreased by ∼60% compared to that of week 1. Similarly, TP in week 2 (*p* = 0.05) significantly decreased by ∼52% compared to that of week 1. However, there were no changes in OP (*p* = 0.200) and CP (*p* = 0.350) between weeks 1 and 2 (Table I). Rapid voiding cycles and decreased threshold pressure are hallmark features of CYP-induced experimental cystitis (Arms and Vizzard, 2011).

#### Correlation of integrated neural activity with bladder pressure following CYP injection

In a representative rat, bladder pressure traces [Fig. 3(a), black traces], sliding RMS [Fig. 3(a), gray traces], and integration of the extracted afferent activity [Fig. 3(a), orange traces] were averaged from saline-induced voids (4–8 voids) and measured prior to CYP injection (days 4 and 6) and post CYP (days 9, 11, and 14). Threshold pressure was indicated for each day on the pressure traces [indicated by a red arrow in Fig. 3(a)].

A non-parametric Kruskal–Wallis test was performed to assess differences between the *R*^2^ of RMS or integrated activity over chronic implantation in *n* = 1 rat challenged with CYP [*n* = 4–10 voids/day; Fig. 3(a)]. Analysis showed there were no significant changes over time in the *R*^2^ of RMS [H_(4)_ = 1.30; *p* = 0.862; *n* = 24 voids from *n* = 1 CYP-challenged rat; Fig. 3(b)] or the *R*^2^ of the integrated activity [H_(4)_ = 2.68; *p* = 0.613; *n* = 24 voids from *n* = 1 CYP-challenged rat; Fig. 3(c)]. A non-parametric paired Wilcoxon signed-rank test was used to assess differences in *R*^2^ of RMS and integrated activity on day 14. Analysis showed *R*^2^ of integrated activity was significantly higher (median: 0.68, *n* = 9 pairs; W = 45, *p* = 0.004) than *R*^2^ of RMS [median: 0.07; Fig. 3(d)].

### Stability of the correlation of integrated neural activity and bladder pressure in all normal and CYP-challenged rats

**FIG. 4.**
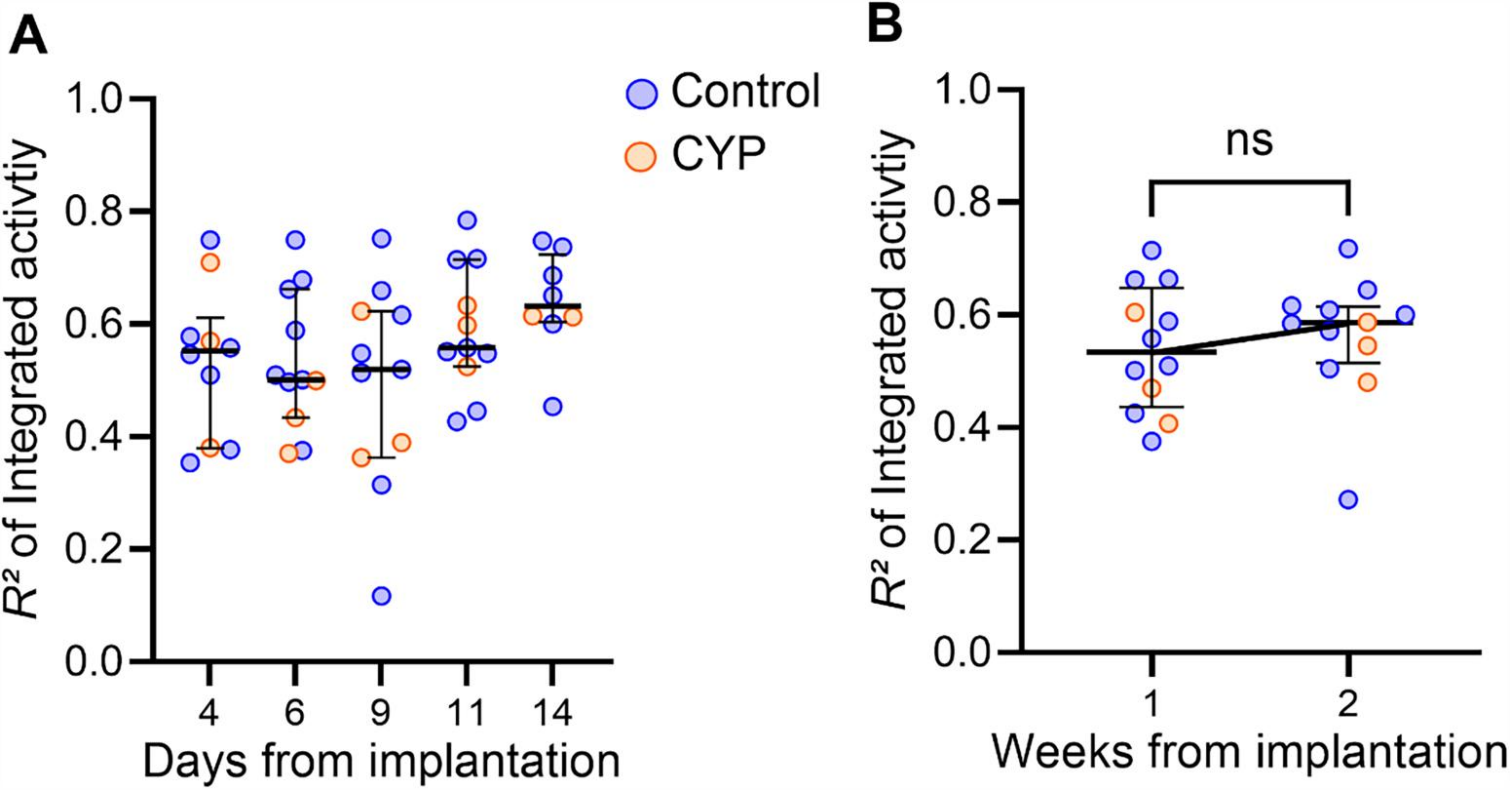
The correlation (*R*^2^) of integrated afferent activity and bladder pressure of all normal and CYP-challenged rats during physiologically evoked saline-induced voiding over 2 weeks of implantation. (a) *R*^2^ from individual control rats (*n* = 9, indicated in the blue line) and CYP-challenged (*n* = 3, indicated by the yellow line) rats. (b) A Wilcoxon analysis shows there were no changes of *R*^2^ of integrated activity at 1 and 2 weeks, *p* = 0.519 following implantation. Data show *R*^2^ of individual rats (*n* = 12), median ± interquartile range (*n* = 12). Significant differences in *R*^2^ between 1 and 2 weeks were accepted as *p* ≤ 0.05.

During 2 weeks of implantation, the correlation between integrated neural activity and bladder pressure (*R*^2^) was relatively stable [Fig. 4 in both control (*n* = 9, blue lines) and CYP-challenged rats (*n* = 3, yellow lines)]. A non-parametric paired Wilcoxon signed-rank test was used to assess differences in *R*^2^ of integrated activity from all rats (control and CYP, total of *n* = 12) between week 1 (average *R*^2^ of days 4 and 6) and week 2 (average *R*^2^ of days 9, 11, and 14). Analysis showed *R*^2^ of integrated activity was no different between week 1 (median *R*^2^: 0.53, *n* = 12 pairs; W = 18, *p* = 0.519) and week 2 [median *R*^2^: 0.59; Fig. 4(b)].

To assess functionality of electrodes during chronic implantation of all rats (*n* = 12), common ground impedance was assessed. Mean common ground impedance during surgery was 9.4 ± 0.7 kΩ. A one-way RM ANOVA (*F*_2,22_ = 9.3, *p* = 0.0012, *n* = 12) and Tukey's *post hoc* multiple comparison test showed impedances remained stable (8.0 ± 0.9 kΩ; *p* = 0.064) at 1 week but significantly increased at 2 weeks (11.2 ± 1.0 kΩ; *p* = 0.0008), compared to intraoperative values.

## DISCUSSION

Neurogenic bladder dysfunction can manifest as an overactive bladder, which causes increases in urinary urgency, frequency, nocturia, and incontinence (Gulur and Drake, 2010) or chronic urinary retention, which causes high bladder pressures, urinary tract infections, vesicoureteral reflux, and kidney damage (Serlin *et al.*, 2018). These conditions require different neuromodulation strategies at different times of the voiding cycle to effectively restore bladder function (Oh *et al.*, 2024; Krhut *et al.*, 2025). As such, a closed-loop system for the treatment of neurogenic bladder dysfunction requires a real-time signal informing on bladder pressure (Park *et al.*, 2018; Oh *et al.*, 2024). Here, we demonstrated that our LINE electrode array and cross correlation analysis technique extracted afferent Aδ activity that strongly correlated with pressures around threshold pressure in awake, freely moving normal animals, and the strength of the correlation was not affected by chronic implantation, i.e., “Time.” Furthermore, data show potential that abnormal afferent Aδ activity, i.e., “Noise” induced following a CYP injection to induce bladder inflammation, does not weaken the correlation. Of significant importance for potential future translation, the same electrode array can be used to both provide neural feedback and deliver stimulation therapy. As such, recording neural activity from the pelvic nerve shows promise to be a useful, objective real-time biomarker for bladder pressure for closed-loop control of neurogenic bladder disorders.

Current neuromodulation bladder control systems are open-loop (Park *et al.*, 2018; Oh *et al.*, 2024; and Krhut *et al.*, 2025). Although results are promising, long-term use of open-loop systems for bladder control shows decrease in efficacy due to neural habituation, risk of tissue damage, and higher power consumption/reduced battery life (Waltz, 2016; Park *et al.*, 2018). As such, a closed-loop system is required to overcome these limitations, delivering temporally precise neuromodulation to induce or arrest a void (Park *et al.*, 2018; Ouyang *et al.*, 2022). A critical component of a closed-loop system is a real-time physiological signal that accurately informs upon the status of the bladder. Some recent studies have shown voiding reflex is triggered by bladder volume rather than pressure (Jaskowak and Danziger 2023). However, as bladder pressure is widely measured in the clinic (Park *et al.*, 2018; Oh *et al.*, 2024; and Krhut *et al.*, 2025), this study focused on the correlation between threshold pressure during the filling phase of micturition and real-time recording of afferent Aδ activity (conduction velocity range: 2.2–10.8 m/s) from the pelvic nerve (Sengupta and Gebhart, 1994b). Threshold pressure is a critical “tipping point” event in the micturition cycle where complex neural reflexes initiate a void. As such, real-time feedback on this event is critical for closed-loop systems to allow appropriate stimulation to be delivered to either arrest or induce a void, depending on the bladder condition. Here, we found in normal rats, integrated afferent Aδ pelvic nerve activity had a strong and stable correlation (*R*^2^: >0.5) to pre-voiding bladder pressure over 2 weeks of implantation. Other studies demonstrate the recording of neural activity or even electroneurograms to inform upon the state of the bladder. For example, a previous study recorded multifiber sensory nerve activity *in situ* from the pelvic nerve of awake mice during cytometry using saline and 0.5% acetic acid. However, no correlation was made with bladder pressure, and the method of recording from sensory activity was not clinically viable (Zvara *et al.*, 2010). Electroneurogram activity of the pudendal nerve was recorded using a tripolar cuff electrode and a cumulative sum algorithm used to detect the onset of hyper-reflexive bladder contractions in anesthetized cats (Wenzel *et al.*, 2005). Other feline studies under anesthesia recorded bladder neuron activity from sacral dorsal root ganglia (DRG) and correlated activity with isovolumetric bladder pressures (Ross *et al.*, 2016; 2018; Sperry *et al.*, 2021). More recently, a Kalman filter-based algorithm estimated real-time bladder pressure from sacral dorsal root ganglia (DRG) neural recordings and triggered sacral root stimulation when pressure increased. During continuous cystometry, closed-loop neuromodulation increased bladder capacity by 13.8% (*p* < 0.001) compared to no stimulation and reduced stimulation time by 57.7% vs continuous stimulation (Ouyang *et al.*, 2022). Although promising, measuring DRG activity is highly invasive and surgically complicated. To our knowledge, this study is the first to show a recording and analysis method that reliably correlates afferent Aδ pelvic nerve activity, specific for class and type of fiber population, with bladder pressure in awake, freely moving rats over a 2-week implantation period.

Here, we report an increase in common ground impedance following chronic implantation of the pelvic nerve, which is in line with our previous pelvic (Payne *et al.*, 2020) and vagus nerve implantation rat studies (Payne *et al.*, 2019; 2022a; 2022b). Increases in common ground impedance are commonly reported during chronic implantation of devices in humans, with notable examples seen with cochlear implants (Paasche *et al.*, 2006) and deep brain stimulation electrodes in humans (Sillay *et al.*, 2013), and are often attributed to the growth of tissue in response to a foreign body (Grill and Thomas Mortimer, 1994). However, in a previous study we showed that there is no significant correlation between impedance, electrically evoked neural threshold, and fibrotic tissue area following chronic implantation of the pelvic nerve in rats (Payne *et al.*, 2025). This suggests, along with the stability of the *R*^2^ over 2 weeks, that increases in common ground impedance have a minimal impact on signal quality during chronic implantation.

In a closed-loop system, bladder biomarkers must reliably reflect the bladder's condition (Oh *et al.*, 2024). Previous efforts, used clinically for detecting “non-neural” bladder biomarkers indicative of bladder events, measure bladder contraction or bladder pressure. Devices monitoring intravesical pressure can regulate bladder function in patients with conditions like overactive bladder or neurogenic bladder dysfunction (Oh *et al.*, 2024). However, long-term use of bladder catheters to measure intravesical pressure is often associated with urinary tract infections and social difficulties. Intravesical electrical stimulation (IVES) is used clinically for the treatment of underactive bladder disorders and measures bladder pressure in real-time and applies stimulation to the detrusor muscle of the bladder wall to trigger contraction (Yu *et al.*, 2025). This method has had some success in reducing post void residual urine volume and increasing voiding volume and urine flow rate. However, treatment efficacy is variable, with some patients reporting pain and bladder irritation, and the placement of a long-term indwelling catheter reportedly leads to urinary infection in 3%–10% of people (Hariati *et al.*, 2019). Alternatively, electromyography (EMG) signals resulting from detrusor muscle and/or urethra sphincter muscles contracting can be recorded to measure bladder contraction using noninvasive electrodes placed on the skin (Wenzel *et al.*, 2005) or via a sensor within a bladder catheter (Majerus *et al.*, 2017; Park *et al.*, 2018). However, low signal-to-noise ratio and accuracy of electrode placement remain challenges for this technique. Bladder pressure can be accurately measured directly via an indwelling catheter inserted via the urethra or suprapubic approach (Yu *et al.*, 2025). In summary, although IVES treatment for bladder disorders is promising, complications arise with long-term indwelling catheters.

This study also showed potential that the stability of the correlation between Aδ activity and bladder pressure was unaffected in rats challenged with CYP as a model of interstitial cystitis and pain syndrome (Kuret *et al.*, 2021). Here, we used a low dose (75–100 mg/kg) to induce chronic cystitis, reflected in the significant reduction of intervoid intervals or increased voiding frequency during saline cystometry, in line with other studies (Arms and Vizzard, 2011). CYP is a chemotherapeutic/alkylating agent metabolized to acrolein to cause damage and inflammation of the urothelial layer of the bladder (Malley and Vizzard, 2002; Augé *et al.*, 2013). Well-characterized rat studies show that following CYP injection, the activity of afferent Aδ mechanoreceptors and usually dormant C-fiber nociceptors is significantly increased in response to pressure changes during voiding (Fowler, 2002; Kuga *et al.*, 2016; 2017). However, despite the presence of increased afferent nociceptor activity, there was no significant effect on the correlation of Aδ activity with bladder pressure during chronic implantation. However, a limitation of this study was that only a modest number of CYP-challenged rats (*n* = 3) were used. These experiments were technically challenging, and so we suggest data show potential that CYP-induced nociceptive activity does not affect the correlation of Aδ activity with bladder pressure. However, additional rat numbers and/or different models of chemically induced interstitial cystitis should be considered for conclusive evidence.

As a step in the translation of closed-loop bladder control, future preclinical studies should consider redesigning the electrode array to allow implantation onto the pelvic nerve of female rats. In this study, only male rats were used for this feasibility study, as the prostate gland serves as a critical structure to suture and secure the electrode array over the pelvic nerve. Furthermore, this study only reports a correlation between *R*^2^ of neural activity and bladder pressure and does not report on prediction accuracy or robustness to artifacts. As such, future translational studies should focus on the prediction of reaching TP based on integrated Aδ activity, particularly during rat movement and non-voiding artifacts, which would make this prediction more challenging.

## CONCLUSION

This study shows recording neural activity as a physiological feedback signal for bladder conditions is feasible and is a promising next step for the translation of a closed-loop system for bladder control. Of significant importance for translation, the same electrode array can be used to both provide neural feedback and deliver stimulation therapy. If successful, closed-loop systems could restore voluntary bladder control and improve quality of life for individuals with neurogenic bladder disorders.

## METHODS

### Animals and housing conditions

Male Sprague-Dawley rats (*n* = 12; aged 8–9 weeks at array implantation, Animal Resource Centre, Western Australia) were used in this study. All animal procedures were approved by St. Vincent's Hospital Animal Ethics Committee and complied with the Australian Code for the Care and Use of Animals for Scientific Purposes (National Health and Medical Research Council of Australia). On arrival, animals were housed 3 per cage and handled daily to habituate animals to human contact for 1 week. Following implantation, rats were housed individually to prevent damage to catheter plugs and the percutaneous pedestal. When housed individually, rats were provided with environmental enrichment, kept under a 12-hr light–dark cycle in a temperature-controlled room, and allowed *ad libitum* access to food and water.

### Anesthesia, analgesia, and euthanasia

Surgical procedures were conducted under isoflurane anesthesia (3% for induction, 1.5–2% for maintenance in 1 l/min oxygen). Analgesia (0.3 ml Carprofen, subcutaneous) and fluids (3 ml/100 g Hartman's Solution, subcutaneous) were provided during surgery. For the first 3 days post-surgery, animals were given fluids twice a day (3 ml/100 g Hartman's Solution, subcutaneous) to help prevent the development of cystitis following bladder catheterization surgery. At the completion of the experiment, animals were humanely killed (350 mg/kg, Lethabarb, intraperitoneal).

### Electrode array design

All animals were chronically implanted with a 2-pair platinum electrode array (electrodes 1–4, E1–E4; made in-house, Bionics Institute). The array was orientated on the pelvic nerve so that electrodes 1 and 2 were closer to the spine, i.e., proximal, while electrodes 3 and 4 were closer to the bladder, i.e., distal (Fig. 1). The distance between electrode pairs (center to center) was 3.25 mm. A ring electrode placed on the cable acted as a subcutaneous return electrode (Payne *et al.*, 2020; 2023).

### Surgical procedures

Detailed methods of the pelvic nerve implantation and bladder catheterization surgery are described in detail in (Fallon *et al.*, 2020; Keast *et al.*, 2020; and Payne *et al.*, 2020). In brief, animals were anesthetized, and under aseptic conditions, the abdominal and pelvic cavities were opened and the left pelvic nerve identified and exposed. The electrode array was placed carefully over the pelvic nerve, ensuring all four electrodes were aligned perpendicular to the pelvic nerve. The array was secured to underlying prostate tissue using sutures (7'0, Ethicon), and care was taken to avoid mechanical damage to neural tissues during implantation. Next, the bladder was catheterized by insertion of a flared polyurethane catheter (PE-10, od: 0.61 mm × id: 0.28 mm, SteriHealth) into the dome of the bladder and secured in the bladder by a purse-string stitch (7'0 silk, Ethicon). The abdominal and pelvic cavities were sutured closed and the catheter tubing routed subcutaneously and exteriorized between the animal's shoulder blades. A pin was placed in the tubing to maintain sterility and prevent leakages of urine. The percutaneous pedestal of the array was sutured to the dorsal-lumbar aspect of the spine, and the skin was closed around it.

### Cystometry

Habituation, cystometry, and analysis of urodynamic parameters were conducted as previously reported (Keast *et al.*, 2020; Payne *et al.*, 2020). In brief, post-surgery the catheter was flushed daily (1 ml of 0.9% sterile saline) to keep the catheter patent. To minimize movement artifacts and the impact of stress on voiding behavior during cystometry, animals underwent 3 days of habituation, which involved spending 1 h inside a Perspex box on an elevated mesh platform while also connected to the infusion pump and recording system. Cystometry was conducted on days 4, 6, 9, 11, and 14 following implantation surgery. Rats were connected to a syringe driver (HA33, Harvard Apparatus, Holliston, MA), and 0.9% sterile saline was continuously infused (9 ml/h) into the bladder. Bladder pressure during filling and voiding was recorded by an inline pressure transducer (MLT0670, AD Instruments, NSW, Australia), amplified (in-house design), and sampled (1k/s; Cereplex Direct, Blackrock® Microsystems, USA). Each testing session lasted for up to 90 min or until 8–10 stable, reproducible voids were generated [Fig. 1(a)] (Andersson *et al.*, 2011; Fraser *et al.*, 2020).

### Cystometry, urodynamic analysis

Cystometry data were analyzed using customized IGOR Pro 9 software (WaveMetrics, USA). Up to four to eight continuous voiding cycles were analyzed to determine standard urodynamic parameters, which included intervoid interval (IVI), the time between the end of one void and the start of another during continuous cystometry; threshold pressure (TP), the bladder's detrusor pressure level that triggers a complex cascade of neural reflexes to cause a void and expulsion of urine; and opening pressure (OP), which is the pressure reached at the first appearance of high-frequency pressure oscillations (HFPOs) during voiding. HFPOs are the rapid phasic contractions of the external urethral sphincter that enable the expulsion of urine in rats (Fraser *et al.*, 2020), and closing pressure (CP), which is the pressure when the expulsion of urine is complete and the external urethral sphincter has closed (Fraser *et al.*, 2020). Pressure relative to baseline was reported for TP, OP, and CP in Table I to allow comparison between previous studies (Wiedmann *et al.*, 2020; Payne *et al.*, 2020).

### CYP injections

Baseline cystometry tests were recorded on days 4 and 6 following chronic implantation. On day 7 rats (*n* = 3) were anesthetized and received a single injection of a low dose of cyclophosphamide (CYP; 75 mg/kg: *n* = 1 or 100 mg/kg: *n =* 2; intraperitoneal, Sigma-Aldrich, St. Louis, MO) to produce urinary bladder inflammation. Cystometry was conducted on days 9, 11, and 14.

### Electrophysiological recordings and neural analysis

#### Impedance measurements

These were performed at 1 and 2 weeks following implantation. To ensure electrode functionality, common ground impedance was measured at implantation by passing biphasic current pulses (100 *μ*A, 100 *μ*s per phase, 50 *μ*s interphase gap) between each electrode and all other electrodes. The peak voltage recorded at the end of the first phase was used to calculate the total impedance (Ohm's law = voltage/current).

#### Cross correlation recording and analysis

As described previously (Payne *et al.*, 2023) pelvic nerve activity physiologically evoked during cystometry was recorded differentially across each bipolar pair of electrodes (E1–E2 and E3–E4). These recordings were amplified (voltage gain: 10^3^ ISO-80, World Precision Instruments, USA), sampled (30k/s; Cereplex Direct, Blackrock® Microsystems, Salt Lake City, UT, USA), and digitally filtered (high pass: 110 Hz, low pass 5000 Hz; Igor Pro 9, Wavemetrics, Inc., Portland, USA). Analysis of the neural recordings was undertaken using customized Igor Pro software [Fig. 1(c); Igor Pro 9, Wavemetrics, USA]. The electrode array [Fig. 1(d)] was used to record physiologically evoked pelvic nerve activity. The cross correlations of large segments of neural recordings (typically 30 s before the opening pressure of the voiding cycle) were used to identify peaks corresponding to different neural classes. Based on the orientation of the electrode array, peaks in the cross correlation at positive lags correspond to efferent activity, while peaks at a negative lag correspond to afferent activity. The peak lag corresponds to the conduction delay between the recording pairs and therefore can be used to estimate conduction velocity. Shorter segments of recordings were then cross-correlated (typical segment length 50 ms, segment overlap 25 ms) to produce a neural heatmap [Fig. 1(e)]. Time-varying activity for different neural populations was extracted from the heatmap by tracking the cross correlation at the appropriate lag [Fig. 1(e)]. The conduction velocity range of Aδ fibers in the pelvic nerve of the rat was accepted as being from 2.2 to 10.8 m/s, which equates to a lag of −1.5 to −0.3 ms [Fig. 1(e)] using an assumed distance of 3.25 mm between electrode pairs [Fig. 1(d)] (Sengupta and Gebhart, 1994a; 1994b). To facilitate comparison of the neural activity with bladder pressure, the root mean square (RMS) of distal neural recording was computed for non-overlapping 0.5 s blocks [Fig. 2(a), gray trace, “RMS activity”], and the extracted neural activity (−1.5 to −0.3 ms lag) was computed for non-overlapping 0.5 s blocks and then integrated over the 30 s prior to TP [Fig. 2(a), blue trace; “integrated Aδ activity”].

### Statistics

Details of each statistical test are stated in the relevant results section. In brief, changes in urodynamic parameters from normal rats over time were analyzed using a parametric paired Student t-test (two-tailed). Urodynamic data from CYP rats (*n* = 3) were not normally distributed, and a Mann–Whitney U test was used. Pearson's correlation tests were used to correlate integrated neural activity or RMS activity with bladder pressure. As *R*^2^ data are typically not normally distributed, a non-parametric Kruskal–Wallis test was performed to assess changes in *R*^2^ of RMS or integrated Aδ activity over time in normal or CYP rats. Differences between the *R*^2^ of RMS and integrated Aδ activity on day 14 following chronic implantation were assessed using a non-parametric paired Wilcoxon signed-rank test. A non-parametric paired Wilcoxon signed-rank test was used to assess differences in *R*^2^ of integrated activity from all rats between week 1 (average of days 4 and 6) and week 2 (average of days 9, 11, and 14). Statistically significant differences were accepted as *p*-values equal to or less than 0.05, and GraphPad Prism 4 (GraphPad Software, USA) or Igor Pro 9 (WaveMetrics, Inc., Portland, USA) was used for all analysis.

## Data Availability

The data that support the findings of this study are openly available in SPARC, [SPARC, 2026].
